# Medical Association State of Florida

**Published:** 1874-02

**Authors:** F. P. Wellford

**Affiliations:** Sec’y & Treas.; Medical Association of the State of Florida


We take pleasure in being able to lay before our readers the proceedings of a meeting of representative medical men of the State of Florida, convened at Jacksonville, on the 14th of January, 1874, for the purpose of organizing a State Medical Association. We congratulate our brethren of Florida upon this forward step, auspiciously taken, and feel full assurance in the prominent character and reputation of the officers chosen, that the foundations of their State Association are being securely and permanently laid. We give the proceedings:

In response to a call issued by the Duval County Medical Society, in November, 1873, a meeting of delegates from different Medical Societies throughout the State of Florida, and individual members of the medical profession, was held at the office of Dr. A. S. Baldwin, in the city of Jacksonville, on the 14th of January, 1874.

Dr. G. W. Betton was elected temporary chairman, and Dr. F. P. Wellford, appointed temporary Secretary.

A committee consisting of Drs. R. P. Daniel and R. B. Burroughs, was appointed by the chairman to examine and report on the credentials, which committee reported as follows:

The committee on credentials for the Convention, for the purpose of organizing a State Medical Society, respectfully report the following named gentlemen present:

| | |
|---|---|
| Dr. A. S. Baldwin,<br>Dr. R. P. Daniel,<br>Dr. F. P. Wellford. | Duval County Medical Society. |
| Dr. J. Peck, | St. John's Co. Medical Society. |
| Dr. G. W. Betton,<br>Dr. R. B. Burroughs. | Leon Co. Medical Society. |
| Dr. E. G. Clay. | Nassau Co. Medical Society. |
| Dr. M. M. T. Hutchingson,<br>Dr. T. W. Carter. | Columbia Co. Med. Society. |
| Dr. T. P. McHenry. | Marion Co. Medical Society. |

The committee would respectfully advise that all the above named gentlemen be considered members of the Convention.

The above report of the committee on credentials was received and unanimously adopted.

On motion of Dr. A. S. Baldwin, an organization was effected, under the title of

"THE MEDICAL ASSOCIATION OF THE STATE OF FLORIDA."

Moved and seconded: That the constitution and by-laws of the Medical Association of Georgia be adopted for the government of this society, until a suitable substitute be provided at its next annual meeting.

A reading of this instrument being called for, Dr. Burroughs, at the request of the chairman, performed that duty, whereupon,

On motion, it was adopted with the exception of those sections referring to the appointment of a Board of Censors, and the specification of the time and place of meeting.

On motion, it was ordered that for the present the office of Secretary and Treasurer shall be held by one individual.

On motion, the chairman appointed a committee of three, composed of Drs. E. G. Clay, R. B. Burroughs and R. P. Daniel, to nominate permanent officers for the Association; which committee reported as follows:

We, the committee on permanent organization, nominate as follows: For President—Dr. A. S. Baldwin, of Duval county. First Vice-President—Dr. G. W. Betton, of Leon county. Second Vice-President, Dr. Robert Harrison, of Nassau county. For Secretary and Treasurer—Dr. F. P. Wellford, of Duval county.

On motion, Dr. Robert Harrison, of Nassau county Medical Society was elected a member of this Association.

The report of the committee on permanent organization was received and adopted, and the following officers duly and unanimously elected for the ensuing year:

Dr. A. S. Baldwin, President.

Dr. G. W. Betton, First Vice-President.

Dr. Robert Harrison, Second Vice-President.

Dr. F. P. Wellford, Secretary and Treasurer.

The Association was then called to order by Dr. A. S. Baldwin, the President elect, who addressed the body in feeling and eloquent terms, in acknowledgement of the compliment conferred.

On motion, it was ordered that a committee of four, to be composed of the permanent officers, be appointed to report a constitution and by-laws to the next annual meeting.

On motion, it was resolved, That the members present pledge their best efforts and influence towards the maintenance of the Association on the most permanent and efficient basis, and to this end do hereby invoke the aid of all regular physicians throughout the State, in the formation of at least one Medical Society in each county thereof.

On motion, the President was authorized to appoint suitable delegates to represent this Association, at the next meeting of the National Medical Association.

On motion, the President was authorized to appoint the time and place for the next annual meeting of this Association, and requested to give timely notice thereof to all the local societies.

The following individuals were nominated and unanimously elected members of the Association:

Dr. A. L. Randolph,

Dr. J. H. Randolph,

Dr. E. T. Sabal,

Dr. J. H. Williams,

Dr. J. D. Palmer,

Dr. J. C. Hill,

Dr. J. D. Fernandez,

Dr. A. J. Wakefield,

Dr. William Shine,

Dr. Andrew Anderson.

Dr. Lewis Pacetti.

Dr. Richard Gardener.

Dr. —— Carn.

Dr. C. — Drew.

On motion, the Association adjourned to meet at the time and place to be specified by the President.

F. P. Wellford, Sec'y & Treas.
Medical Association of the State of Florida.

